# Caste-Specific and Sex-Specific Expression of Chemoreceptor Genes in a Termite

**DOI:** 10.1371/journal.pone.0146125

**Published:** 2016-01-13

**Authors:** Yuki Mitaka, Kazuya Kobayashi, Alexander Mikheyev, Mandy M. Y. Tin, Yutaka Watanabe, Kenji Matsuura

**Affiliations:** 1 Laboratory of Insect Ecology, Division of Applied Biosciences, Graduate School of Agriculture, Kyoto University, Kyoto, 606–8502, Japan; 2 Ecology and Evolution Unit, Okinawa Institute of Science and Technology Graduate University, Okinawa, 904–0495, Japan; United States Department of Agriculture, Beltsville Agricultural Research Center, UNITED STATES

## Abstract

The sophisticated colony organization of eusocial insects is primarily maintained through the utilization of pheromones. The regulation of these complex social interactions requires intricate chemoreception systems. The recent publication of the genome of *Zootermopsis nevadensis* opened a new avenue to study molecular basis of termite caste systems. Although there has been a growing interest in the termite chemoreception system that regulates their sophisticated caste system, the relationship between division of labor and expression of chemoreceptor genes remains to be explored. Using high-throughput mRNA sequencing (RNA-seq), we found several chemoreceptors that are differentially expressed among castes and between sexes in a subterranean termite *Reticulitermes speratus*. In total, 53 chemoreception-related genes were annotated, including 22 odorant receptors, 7 gustatory receptors, 12 ionotropic receptors, 9 odorant-binding proteins, and 3 chemosensory proteins. Most of the chemoreception-related genes had caste-related and sex-related expression patterns; in particular, some chemoreception genes showed king-biased or queen-biased expression patterns. Moreover, more than half of the genes showed significant age-dependent differences in their expression in female and/or male reproductives. These results reveal a strong relationship between the evolution of the division of labor and the regulation of chemoreceptor gene expression, thereby demonstrating the chemical communication and underlining chemoreception mechanism in social insects.

## Introduction

Animals perceive chemical signals in their environment using chemical senses (olfaction and gustation), mainly to locate potential food sources, detect predators, and to receive chemical cues in social interactions [1]. A chemical signal used to communicate among members of the same species is called a pheromone [2]. The sophisticated colony organization of eusocial insects is primarily maintained through the utilization of pheromones, which are also likely involved in all social activities including foraging, sexual behavior, defense, nestmate recognition, and caste regulation [3]. The extraordinary diversity of pheromones is the evolutionary consequence of the powerful and flexible way that the chemoreception system is organized [1].

Division of labor is one of the most prominent features of colony behavior in social insects [4]. Individuals in these societies interact via a complex web of chemical signals. Studies of division of labor have typically been concerned with the integration of individual worker behavior into colony-level task organization, and with the question of how it is regulated by chemical communication [5]. The regulation of complex social interactions requires intricate systems of chemical communication [6], and the evolution of division of labor should be strongly related to the evolution of chemoreception systems. It is reasonably predicted that individuals engaging in different tasks can differ in their sensitivity to various chemicals, and thus may have different sets of chemoreceptors. However, the relationship between caste differentiation and the differential expression of chemoreceptor genes is not well understood.

**Fig 1:**
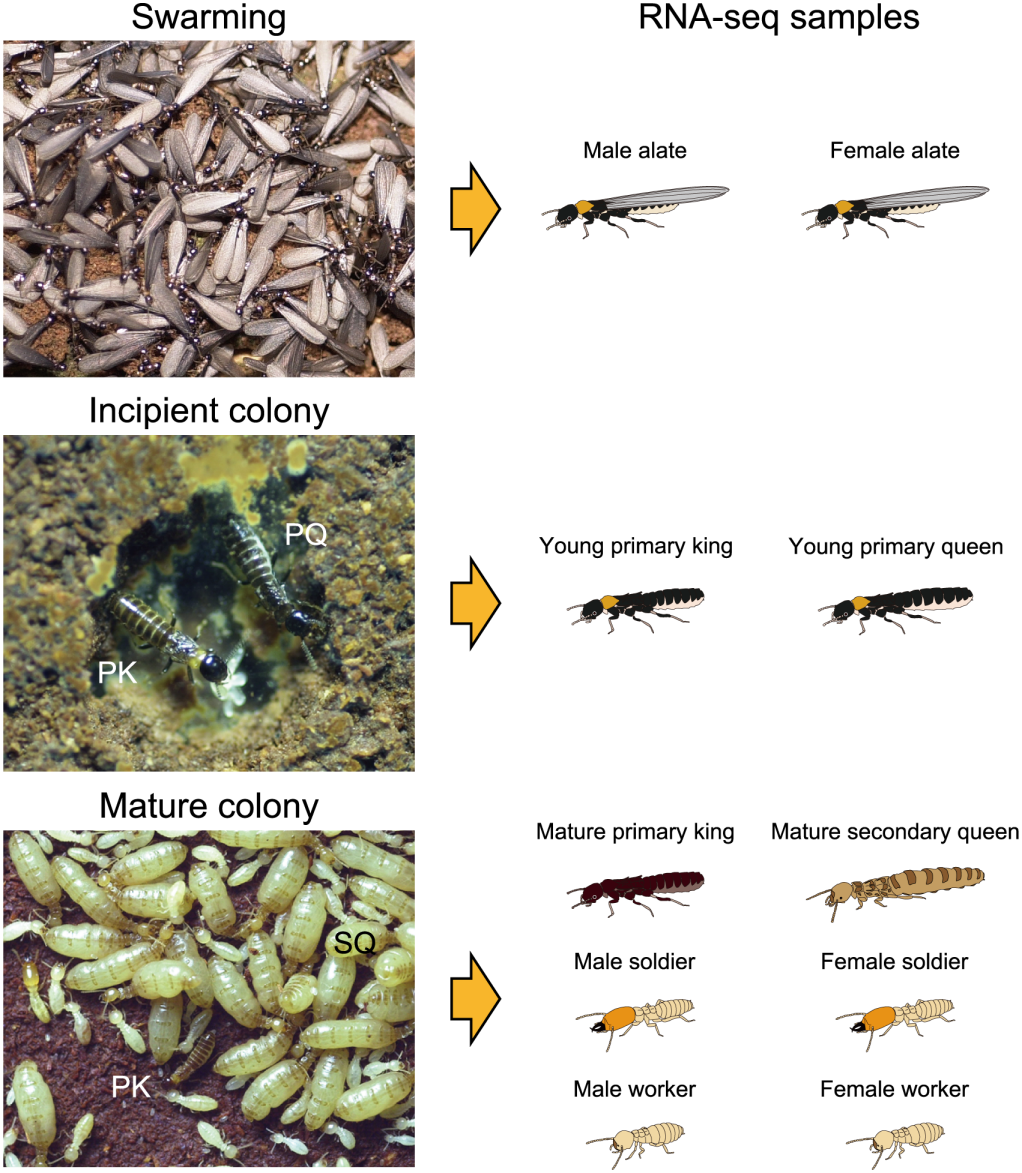
Life history of *R*. *speratus* and RNA-seq samples. Each colony of *R*. *speratus* produces innumerable alates in spring. After swarming, a pair of male and female alates establishes a new colony, and starts to produce offspring sexually as a primary king (PK) and a primary queen (PQ). As the PQ senesces, secondary queens (SQ) produced asexually by the PQ differentiate within the colony and supplement egg production, eventually replacing the PQ. For RNA sequencing (RNA-seq) analysis, we obtained alates from three swarming colonies, and the young PK and PQ were obtained from three incipient colonies artificially established by the same colonies. Mature PK, mature SQ, and both sexes of soldiers and workers were obtained from four large field colonies.

Eusociality in Isoptera (termites) converges along many lines of colony organization and social behavior in eusocial Hymenoptera (ants, bees, and wasps). Unlike social Hymenoptera, however, termites of both sexes are diploid, and their societies consist of both sexes of helpers (workers and soldiers) and reproductives (kings and queens). In termites, pheromones produced by reining queens inhibit the differentiation of new reproductive individuals [7–9]. Recently, a termite queen pheromone consisting of a volatile blend of n-butyl-n-butyrate (nBnB) and 2-methyl-1-butanol (2M1B) was identified in the subterranean termite *Reticulitermes speratus* [10]. Moreover, the breeding system of *R*. *speratus* is characterized by asexual queen succession (AQS) (i.e., queens produce their neotenic replacements asexually, but use normal sexual reproduction to produce other colony members) [11, 12] (Fig 1). The perception of the inhibitory queen pheromone may be involved in the underling mechanism through which asexually produced daughters have a developmental priority to be neotenic queens [13]. Therefore, *R*. *speratus* provides an ideal system to understand how chemical communications regulate the sophisticated caste system in termites. There has been a growing interest in the chemoreception system of termites. Recently, the genome of a dampwood termite *Zootermopsis nevadensis* was sequenced, which first provided the list of chemoreceptor genes in termites [14]. However, there is no information on the differential expression of chemoreceptor genes among castes and between sexes in termites.

**Fig 2:**
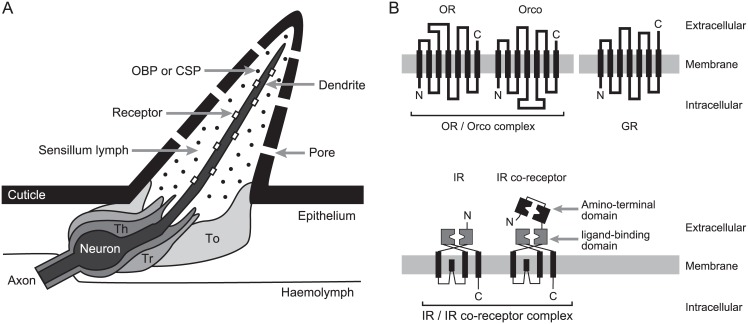
Details of chemoreception. (A) General structure of an insect sensillum (modified from [15]). In insects, odorants are detected by the dendrites of neuron that are housed within chemosensilla mainly on antennae and maxillary palps, and contact chemicals are detected mainly on labial palps and tarsi. The neurons are surrounded by accessory cells (To: tormogen, Th: thecogen, and Tr: trichogen cells). The dendrites of neurons are bathed in aqueous sensillar lymph that protects them from dehydration. After entering the sensillum through cuticular pores, the chemicals are transported by odorant-binding proteins (OBPs) or chemosensory proteins (CSPs) to reach the chemoreceptor on the membrane of dendrite as crossing the lymph. Each chemical binds to a corresponding chemoreceptor, thereby a chemical signal in the environment is converted into an electrical signal that can be interpreted by the insect nervous system. (B) Schematic diagrams of odorant receptor (OR), gustatory receptor (GR), and ionotropic receptor (IR) (modified from [16–18]). Insect ORs and GRs are seven-transmembrane domain proteins with a reversed membrane topology (intracellular N-terminus) compared to vertebrate ORs. The OR determining ligand specificity forms heteromers with a highly conserved odorant receptor co-receptor (ORCO) in insects. IRs have recently been discovered as another class of receptors involved in chemoreception in *D*. *melanogaster*. IR forms heteromers composed of the compound-specific receptor and the co-receptor.

Here, we assembled and analyzed transcriptomes from next-generation sequencing of all castes of *R*. *speratus* including the mature primary king, secondary queen, and both sexes of alates, founders (young primary kings and queens), soldiers, and workers to identify chemoreception-related genes (Fig 2) and compare their expression among castes and sexes. From the obtained mRNA library, the candidate genes of the odorant receptor (OR), gustatory receptor (GR), ionotropic receptor (IR), odorant-binding protein (OBP), and chemosensory protein (CSP) were annotated. Subsequently, the expression of each chemoreception-related gene was compared among castes and between sexes.

## Results

### Transcriptome sequencing and *de novo* transcriptome assembly

Illumina sequencing of all castes and sexes in *R*. *speratus* yielded a total of 729 M read pairs with an average length of 93 bp for each short read, and the percentage of GC content was 41%. The transcriptome was assembled *de novo* with Trinity [19, 20] and yielded a total of 856 Mbp of mRNA sequences, 1,144,272 contigs with a minimum length of 201 bp, a median length of 360 bp, a maximum length of 35,267 bp, a N50 value of 1,296 bp, and an average length of 748 bp (S1 Fig and Table 1).

**Table 1:** Summary statistics of assembly.

Assembly assessment parameters	Transcriptional products	Translation products
Total length	856,112,139 bp	76,684,150 aa
Number of gene products	1,144,272	156,276
Average length	748 bp	491 aa
Median length	360 bp	381 aa
Maximum length	35,267 bp	11,369 aa
Minimum length	201 bp	100 aa
N50 length	1,296 bp	584 aa
GC content	41%	-

Using transcriptsToOrfs, 156,276 ORFs were detected with a total length of 76,684,150 amino acids (aa), an average length of 490.70 aa, a median length of 381 aa, a maximum length of 11,369 aa, a minimum length of 100 aa, and a N50 length of 584 aa (Table 1). Based on the subsequent homology search of the known amino acid sequence data from *Zootermopsis nevadensis* [14], *Acyrthosiphon pisum* [21], *Apis mellifera* [22], *Tribolium castaneum* [23], *Bombyx mori* [24], *and Drosophila melanogaster* [25], 10,238 protein-coding transcripts were inferred in *R*. *speratus*.

### Odorant receptor

We found 22 putative ORs through a blast search with the amino acid sequences of ORs in various insect species in the query (S2 Table); all were classified as being in the superfamily of 7-transmembrane receptors (BLASTP, NCBI). RsORCO showed higher sequence similarity to the ORCO [15] of *Z*. *nevadensis* (91%), *M*. *caryae* (64%), *A*. *mellifera* (60%), *B*. *mori* (59%), *T*. *castaneum* (64%), *Dendroctonus ponderosae* (64%), *D*. *melanogaster* (72%), *Anopheles gambiae* (73%), *Aedes aegypti* (72%), and *Locusta migratoria* (63%). The phylogeny inferred from the ORCO amino acid sequences was consistent with the previously known topology of the insect order (S3 Fig). The ligands of other receptors remain unknown.

**Fig 3:**
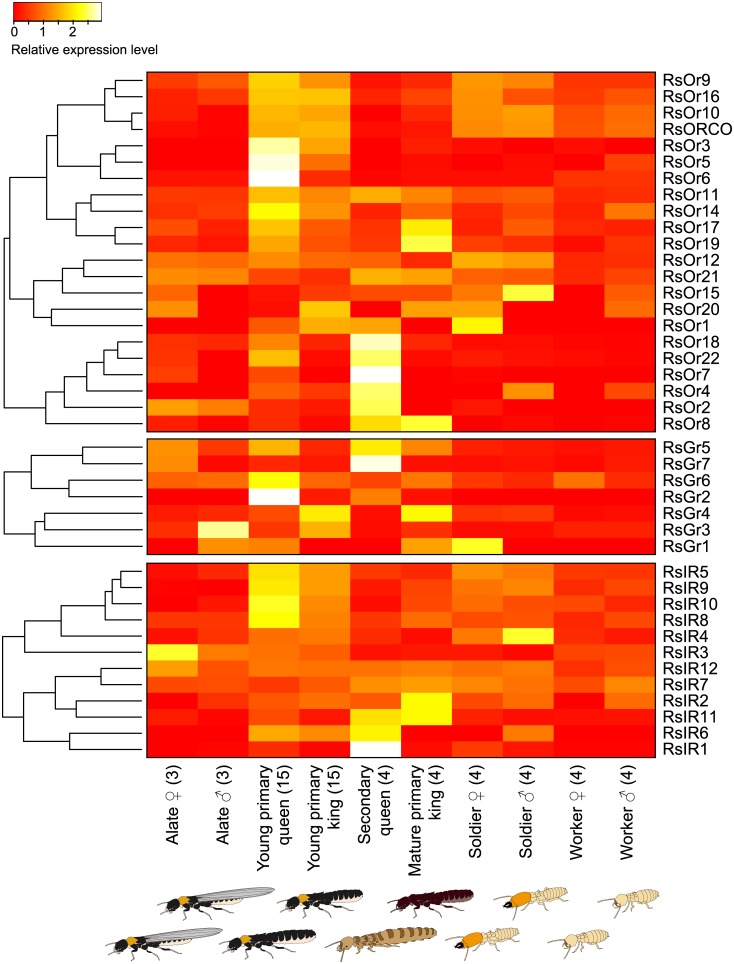
Differential expression of chemoreceptors among castes. The heatmaps exhibit the differential expression of 22 OR, 7 GR, and 12 IR genes among termite castes. Relative expression level indicates the mean normalized Count per Million (CPM), ranging from red (scaled expression of 0) to white (scaled expression of 2.5). The tree at the left corresponds to hierarchical clustering of cluster-averaged expression. Numbers in parenthesis after caste names refer to the numbers of biological replication. Ten individuals were pooled for each sex of worker and soldier to obtain sufficient amount of RNA, while single individuals were used for RNA extraction of the other castes.

**Fig 4:**
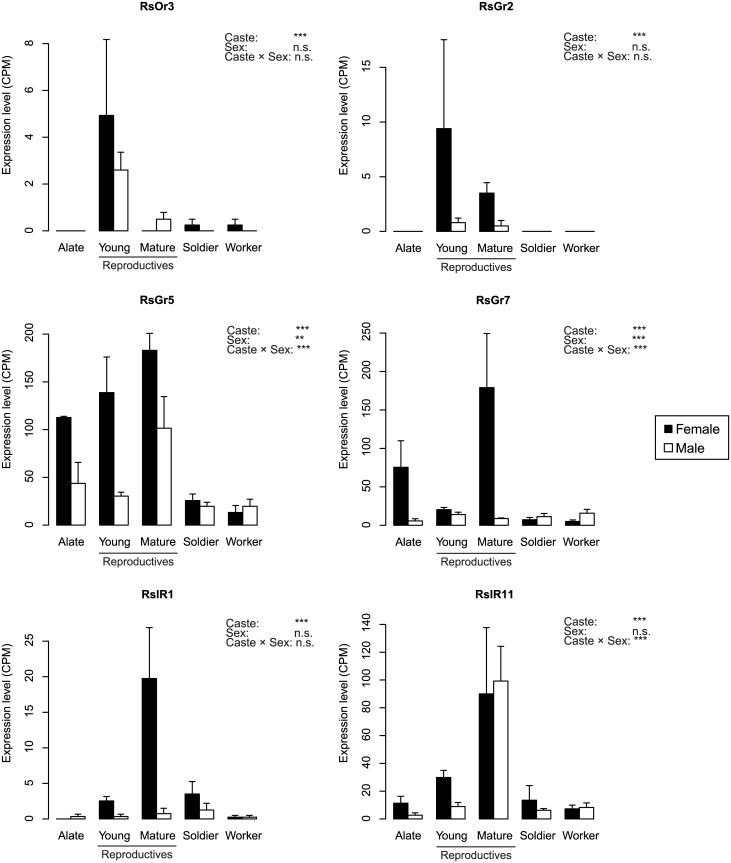
Characteristic expression patterns of ligand-predicted chemoreceptors. Comparison of the mean CPM of six receptor transcripts among castes and between the sexes (black: female, white: male) of each caste. Young reproductives refer to young PQs and PKs. Mature reproductives indicate mature secondary queens and mature PKs. Error bars denote standard errors. Results of statistical analyses for each gene expression are shown in the upper right side of each graph (n.s.: not significant, *: False Discovery Rate (FDR) < 0.05, **: FDR < 0.01, ***: FDR < 0.001).

The expression level of RsOr3 was zero in both sexes of alates, secondary queens, and male workers and soldiers (caste: false discovery rate (FDR) < 0.001; Figs 3 and 4 and S3 Table). RsOr10 was commonly expressed in all of the castes except the male alates and secondary queens (caste: FDR < 0.001, caste × sex: FDR < 0.05; S3 Table). RsORCO was more highly expressed in young primary kings and queens, soldiers and workers than other castes (caste: FDR < 0.001; Fig 3 and S2 Fig and S3 Table). Some ORs were more highly expressed in reproductive castes than in other castes (Figs 3 and 4 and S2 Fig). RsOr5 was expressed more in young primary kings and queens (caste: FDR < 0.001; S3 Table), whereas RsOr8 was expressed more in mature primary kings and secondary queens (caste: FDR < 0.001, sex: FDR < 0.05; S3 Table). Four ORs (RsOr2, RsOr7, RsOr18, and RsOr22) were expressed more in secondary queens (caste: FDR < 0.001; S3 Table), whereas RsOr9 and RsOr16 were commonly expressed but showed differential expression among castes (caste: FDR < 0.001, S3 Table).

### Gustatory receptor

Seven GRs were predicted through a blast search with the GR sequences of *D*. *melanogaster*, *Z*. *nevadensis*, and *T*. *castaneum* (S2 Table), and were classified as being part of the 7-transmembrane superfamily (BLASTP, NCBI). RsGr2 was only expressed in reproductive castes, especially in young primary queens and secondary queens (caste: FDR < 0.001; Fig 4 and S3 Table). RsGr5 and RsGr7 were expressed more in female reproductive castes (RsGr5: caste: FDR < 0.001, sex: FDR < 0.01, caste × sex: FDR < 0.001, RsGr7: caste: FDR < 0.001, sex: FDR < 0.001, caste × sex FDR < 0.001; Fig 4 and S3 Table). Three GRs (RsGr3, RsGr4, and RsGr6) were commonly expressed in all of the castes (Fig 3 and S4 Fig), but only RsGr3 showed no differential expression among castes (caste: n.s., sex: n.s., caste × sex: n.s.; S3 Table). RsGr1 was only expressed in male alates, young primary queens, mature primary kings, and female soldiers (caste × sex: FDR < 0.05; S3 Table).

### Ionotropic receptor

We found 12 putative IRs based on their sequence similarity to those of *D*. *melanogaster* (S2 Table); all were classified as being part of the periplasmic binding protein superfamily (BLASTP, NCBI). RsIR1 showed secondary-queen-biased expression (caste: FDR < 0.001; Fig 4 and S3 Table), and RsIR11 showed higher expression in secondary queens and mature primary kings (caste: FDR < 0.001, caste × sex: FDR < 0.001; Fig 4 and S3 Table). Seven IRs (RsIR3, RsIR5, RsIR6, RsIR7, RsIR8, RsIR9, and RsIR10) had differential caste expression (caste: FDR < 0.001; S5 Fig and S3 Table), and in particular, RsIR10 also indicated a caste-sex interaction (caste × sex: FDR < 0.001; S3 Table). RsIR2 and RsIR4 were equally expressed in all of the castes (caste: n.s.; S5 Fig and S3 Table).

### Odorant-binding protein

**Fig 5:**
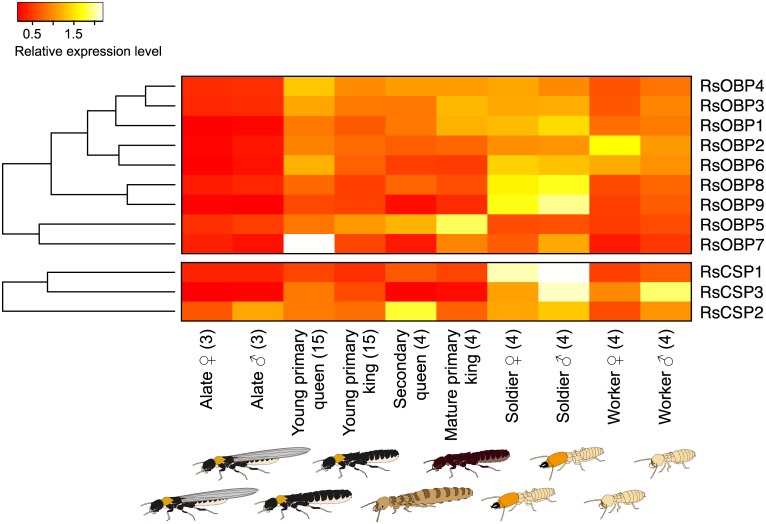
Differential expression of OBP and CSP among castes. The heatmaps exhibit the differential expression of nine OBPs and three CSPs transcripts among termite castes. Abbreviations are referred to in Fig 3. Relative expression level indicates the mean CPM, ranging from red (scaled expression of 0) to white (scaled expression of 2). Tree at the left correspond to hierarchical clustering of cluster-averaged expression.

Nine OBPs were predicted, which resembled the sequences of OBPs of *Periplaneta americana*, *D*. *melanogaster*, *A*. *gambiae*, *T*. *castaneum*, and *Rhodnius prolixus* (Fig 5, S2 Table). Two OBPs (RsOBP8, and RsOBP9) were expressed more in soldiers (caste: FDR < 0.001; S6 Fig, S3 Table). Six OBPs (RsOBP1, RsOBP2, RsOBP3, RsOBP4 RsOBP6 and RsOBP7) were expressed in all of the castes except alates (caste: FDR < 0.001; S6 Fig and S3 Table), whereas the expression level of RsOBP5 was higher in mature primary kings than in other castes (caste: FDR < 0.001; S6 Fig and S3 Table).

### Chemosensory protein

Three CSPs were predicted, and these showed sequence similarity to those of *Holotrichia oblita*, *Adelphocoris lineolatus*, *T*. *castaneum*, and *B*. *mori* (S2 Table). RsCSP1 was expressed more in soldiers than in other castes (caste: FDR < 0.001; Fig 5, S6 Fig and S3 Table). RsCSP2 and RsCSP3 showed significant expression among castes (caste: FDR < 0.001, caste × sex: FDR < 0.01; Fig 5, S6 Fig and S3 Table).

### Age-related alternation of gene expression levels

Among the 53 annotated chemoreception-related genes, 26 receptors (13 ORs, 6 GRs and 7 IRs) and 7 binding proteins (7 OBPs and 2 CSPs) showed significant differences among the reproductives of different age classes (S4 Table). In male reproductives (male alates, young primary kings, and mature primary kings), young primary kings showed higher expression in 11 receptors (RsOr3, RsOr5, RsOr9, RsOr10, RsORCO, RsOr16, RsIR4, RsIR5, RsIR6, RsIR9, and RsIR10) and 2 binding proteins (RsOBP9 and RsCSP3) than in other age classes (FDR < 0.05; S2, S5 and S6 Figs and S4 Table), and mature primary kings showed the highest expression in 5 receptors (RsOr8, RsOr17, RsOr19, RsGr5, RsIR11) and 4 OBPs (RsOBP1, RsOBP3, RsOBP5, RsOBP7) (FDR < 0.01, Fig 4, S6 Fig and S4 Table). In female reproductives, young primary queens showed higher expression in 9 receptors (RsOr3, RsOr5, RsOr12, RsORCO, RsGr2, RsGr5, RsIR5, RsIR9, RsIR10) and 6 binding proteins (RsOBP1, RsOBP4, RsOBP6, RsOBP7, RsOBP9 and RsCSP3) than in female alates (FDR < 0.05, S2, S4, S5 and S6 Figs and S4 Table).

## Discussion

Using our RNA-seq dataset, which included RNA assayed from individuals of different castes, sexes, and ages of *R*. *speratus*, we detected 156,276 ORFs. Based on the subsequent homology search of the known amino acid sequence data of insects (*Z*. *nevadensis*, *A*. *pisum*, *A*. *mellifera*, *T*. *castaneum*, *B*. *mori*, *and D*. *melanogaster*), we inferred 10,238 protein-coding transcripts from the RNA-seq data; among the 10,238 genes, 53 were annotated as chemoreception-related genes consisting of 22 ORs, 7 GRs, 12 IRs 9 OBPs, and 3 CSPs.

Termite societies are characterized by a highly sophisticated caste system with distinct morphological and behavioral differences. Workers perform a variety of tasks including brood and royal care, foraging, nest construction and hygienic activities. In contrast, soldiers and royals are engaged exclusively in nest defense and reproduction, respectively. This indicates that chemosensory demands would be different among castes. As expected, 81% (43/53) of chemoreception-related genes showed significant caste-specific differences (S3 Table), suggesting adaptation of chemoreception systems for efficient task allocation. The functional significance of the caste-specific expression patterns remains unknown until the ligands of these receptors are identified by future studies.

Among the 53 chemoreception-related genes, 33 genes (62%) showed significant age-dependent differences in their expression in female and/or male reproductives (S4 Table). In female reproductives, 15 genes showed significantly higher expression levels in young primary queens than in female alates, but 5 receptor genes showed the opposite pattern (FDR < 0.01, Fig 4, S2 and S5 Figs and S4 Table). In male reproductives, thirteen genes had the highest expression in young primary kings, and nine genes in mature primary king, but four genes were most highly expressed in male alates (FDR < 0.05, S2, S4, S5 and S6 Figs and S4 Table). These age-dependent expression patterns indicate that many chemoreception-related genes increase expression after the colony is founded.

ORCO interacts with ligand-specific ORs and forms heterodimeric complexes that are essential for trafficking of ORs [26, 27]. ORCO is strikingly well conserved across insect species [28, 29]. In this study, RsORCO showed a high degree of sequence similarity with ORCOs of other insects suggesting an evolutionarily conserved function (S3 Fig and S2 Table). The expression of RsORCO was much higher in individuals engaged in social labor (workers, soldiers and founding pairs) than in those of dispersal stage (alates) and mature reproductive stage (mature king and queens), implying that RsORCO expression may reflect the activity of ORs as a whole. It is important to note that the expression of RsORCO was lower than the total expression of ORs and similar patterns can be seen in the data of the silk moth *Bombyx mori* [30] and the red flour beetle *Tribolium castaneum* [31]. Hence, the quantitative relationship between the expression of ORCO and the total expression of ORs remains to be explored in the future studies.

Our analysis of the chemoreceptor genes in the termite has begun to reveal the relationship between division of labor and expression of chemoreceptor genes. In this study we performed the RNA-seq on whole bodies of termites. Therefore, the number of reported chemoreceptor genes might be underestimated, leaving the possibility for future works to find more chemoreceptors showing caste-, sex- or age-specificity. These results provide new insights into the roles of chemoreceptors in the division of labor, and provide a foundation for exploring the mechanism and evolution of chemoreceptor polyphenism in termites.

## Materials and Methods

### Termites

To evaluate the expression of chemoreception-related genes, we conducted RNA sequencing (RNA-seq) transcriptome analysis of male and female alates, young primary kings and queens, mature primary kings and mature secondary queens, and male and female soldiers and workers of the subterranean termite *R*. *speratus* (Fig 1). To obtain alates, we collected three colonies (colony code: TA130412C, TA130412E, and HI130508N) from secondary forests in Kyoto prefecture, Japan, in the swarming season from April to May 2013 (S1 Table). The alates extracted from the nests were separated by sex, and maintained in Petri dishes containing moist filter paper until they shed their wings. Then, a male and female were randomly selected from each colony and placed in a 90 mm Petri dish that contained mixed sawdust bait blocks [32]; five nestmate pairs were made for each colony. The Petri dishes were kept at 25°C under constant darkness. After 6 months, the nests in bait blocks were dissected to collect young primary queens and kings from the incipient colonies (S1 Table).

To obtain kings and queens from mature colonies of *R*. *speratus*, we collected termite nests from secondary forests in Kyoto and Shiga prefecture, Japan, during the reproductive season from July to October 2013. We found royal chambers of four colonies (HI130717B, ZE130827B, ZE130827H, and YO131010A), and carefully extracted kings and queens together with workers and soldiers (S1 Table). All of the kings were primary kings and all of the queens were secondary queens. Workers and soldiers were separated by sex based on the caudal sternite configuration using a stereoscope.

No specific permits were required for the described field studies and no specific permissions were required for the locations/activities for termite sampling because they are public lands and are not privately owned or protected in any way. These field studies did not involve endangered or protected species.

### RNA extraction and Illumina sequencing

Total RNA was extracted from the whole body of each individual of each reproductive caste (alates, kings, and queens) using an RNeasy mini kit (Qiagen), using the standardized instructions from the manufacturer. For workers and soldiers, we pooled 10 individuals of each sex to extract a sufficient amount of RNA for analysis, and performed RNA-seq analysis on a total of 60 samples (DDBJ BioSample ID: SAMD00026264-SAMD00026323). Following previously described procedures [33] for first-strand cDNA synthesis, 9 μL mixtures containing more than 50 ng of each total RNA sample and 20 pmol polyT-START primer (AATTGCAGTGGTATCAACGCAGAGCGGCCGCTTTTTTTTTTTTTTTTTTTTTTTTTTTTTVN) were incubated for 3 min at 65°C, and then chilled on ice for 3 min. Then, the following were added to each sample: 4.0 μL 5x first-strand synthesis buffer (Invitrogen), 1.0 μL 10 mM dNTP mix (Promega), 2.0 μL 0.1 M DTT (Invitrogen), 2.0 μL 12 μM template-switching RNA primer (AAGCAGUGGUAUCAACGCAGAGUACAUGGG), and 1 μL SuperScript II reverse transcriptase (Invitrogen). The samples were incubated for 1 h at 42°C, and reactions were terminated by heating at 65°C for 15 min. Then, the samples were placed on ice and diluted with 80 μL TE buffer prior to cDNA amplification. The cDNA libraries were prepared for second-strand synthesis and amplification. The reaction mixtures were composed of 5 μL diluted first strand cDNA, 10 μL 5x Phusion HF buffer (Finnzymes), 1 μL 10 mM dNTP mix (Promega), 1 μL primer pair (10 μM each; START: CGCCAGGGTTTTCCCAGTCACGACAATTGCAGTGGTATCAACGCAGA and TS_LONG: CTTGTAGGTTAAGTGGAGAGCTAACAATTTCACACAGGAAAGCAGTGGTATCAACGC), and 0.5 μL Phusion High-Fidelity DNA polymerase (Finnzymes), and were heated at 98°C for 30 s followed by 15 cycles of 10 s at 98°C, 6 min at 68°C, and finally 10 min at 72°C. The PCR products were purified with Dynabeads MyOne Carboxylic acid (Invitrogen) and 16% PEG6000 with 0.9 M NaCl and 10 mM Tris-HCl (pH 6), and 12 μL EB buffer was used during the final elution. Tagmentation followed the Nextera XT DNA Sample Prep Kit (illumina) protocol for Hiseq. In January 2014, paired-end 100-cycle multiplex sequencing was performed using the Illumina HiSeq 2000 at the Okinawa Institute of Science and Technology Graduate University. RNA sequence data was deposited in the DNA Data Bank of Japan (DDBJ) under the BioProject “Royal epigenetics in the termite *Reticulitermes speratus*” (PRJDB3531), which contains links and access to insect sampling data through the BioSample SAMD00026264-SAMD00026323 and the Sequence Read Archive DRR030795-DRR030854.

### Data processing and *de novo* transcriptome assembly

The raw sequencing reads were trimmed by removing adapter sequences. Preprocessing was performed in the cloud-computing based analytical platform, DDBJ Read Annotation Pipeline. Bases with a quality score of less than 20 were trimmed from the 5’ and 3’ ends of each read. After trimming, reads with a high percentage (> 30%) of low quality bases (< 15) and short reads (< 25 bp) were discarded, and the remaining reads were used for the assembly. The remaining reads from all of the samples were assembled *de novo* using Trinity version trinityrnaseq_r2012–04–27 [19, 20], which generates transcriptome assemblies from short read sequences using the de Bruijn graph algorithm. The parameters selected to run Trinity were all default parameters (k-mer length = 25-mers) except max_reads_per_loop, which was set to 1,500,000. Using transcriptsToOrfs (version 0.0.2), we searched open reading frames (ORFs) in the assembled sequences, and gained predicted amino acid sequences.

### Protein-coding prediction

We converted the predicted amino acid sequences into a searchable database using the formatdb tool of the BLAST+ v2.2.29 toolkit (National Center for Biotechnology Information (NCBI), http://www.ncbi.nlm.nih.gov/). To predict gene models using the homology-based method, protein sequences from *Z*. *nevadensis* (v2.2, http://termitegenome.org), *A*. *pisum* (v2.1b, http://www.aphidbase.com), *A*. *mellifera* (v3.2, http://hymenopteragenome.org/beebase), *T*. *castaneum* (v3.0, http://beetlebase.org), *B*. *mori* (v2.0, http://silkworm.genomics.org.cn), and *D*. *melanogaster* (v6.02, http://flybase.org) were used for BLAST queries with an E-value cutoff of 1e−100.

### Annotation of chemoreceptor and binding proteins

Peptide sequences of chemoreceptors and binding proteins of the following species were obtained from NCBI, and used as BLAST queries for our peptide database with an E-value cutoff of 1E−7: *Z*. *nevadensis* [14], *P*. *americana* [34], *T*. *castaneum* [31, 35, 23], *H*. *oblita* [36], *D*. *ponderosae* [37], *A*. *mellifera* [38, 39], *D*. *melanogaster* [17, 40–43], *A*. *gambiae* [44], *A*. *aegypti* [45], *A*. *lineolatus* [46], *R*. *prolixus* [47], *L*. *migratria* [48], and *B*. *mori* [30, 49, 50]. The peptide sequences of ORs of *M*. *caryae* were translated from the nucleotide sequences [51] using the Nucleotide Sequence Translation tool (EMBL-EBI, http://www.ebi.ac.uk/Tools/st/).

### Abundance estimation and differential expression analyses

Gene expression levels were estimated by the RSEM version 1.2.8 [52] separately for the filtered reads from each sample. Raw read counts data produced by RSEM were normalized using the Trimmed Mean of M-value (TMM) normalization method [53] and were used for differential expression analyses among castes and between sexes using edgeR package (v3.4.2) [54]. All of the statistical analyses were performed using the R package (v3.0.3) and heatmaps were generated with heatmap.2 in gplots package.

## Supporting Information

S1 Fig Length frequency distribution of *R*. *speratus* contigs. Histogram presentation of sequence-length distribution for significant matches that was found. The horizontal axis indicates sequence sizes from 200 to ≥3000 nucelotides. The vertical axis indicates the number of contigs for each size.
(EPS)

S2 Fig Expression levels of ORs other than RsOr3. Comparison of the mean CPM of OR transcripts among castes and between the sexes (black: female, white: male) of each caste. Young reproductives refer to young PQs and PKs. Mature reproductives indicate mature secondary queens and mature PKs. Error bars denote standard errors. Results of statistical analyses for each gene expression are shown in the upper right side of each graph (n.s.: not significant, *: FDR < 0.05, **: FDR < 0.01, ***: FDR < 0.001).
(EPS)

S3 Fig Phylogenetic tree of ORCO sequences from various insects. The tree was constructed based on the ORCO sequence of *R*. *speratus* and that of other insect species obtained from the protein database of NCBI. The evolutionary history was inferred using the Maximum Likelihood method based on the JTT matrix-based model [55]. The tree with the highest log likelihood (-12311.7185) is shown. The bootstrap values (1,000 resampling) are shown on the branches. Initial tree(s) for the heuristic search were obtained by applying the Neighbor-Joining method to a matrix of pairwise distances estimated using a JTT model. The tree is drawn to scale, with branch lengths measured in the number of substitutions per site. The analysis involved 84 amino acid sequences. All of the positions containing gaps and missing data were eliminated. There were a total of 382 positions in the final dataset. Evolutionary analyses were conducted in MEGA6 [56]. Branch lengths are proportional. The number written before each scientific name is accession numbers.
(EPS)

S4 Fig Expression levels of GRs other than RsGr2, RsGr5, and RsGr7. Comparison of the mean CPM of GR transcripts among castes and between the sexes (black: female, white: male) of each caste. Young reproductives refer to young PQs and PKs. Mature reproductives indicate mature secondary queens and mature primary kings. Error bars denote standard errors. Results of statistical analyses for each gene expression are shown in the upper right side of each graph (n.s.: not significant, *: FDR < 0.05, **: FDR < 0.01, ***: FDR < 0.001).
(EPS)

S5 Fig Expression levels of IRs other than RsIR1 and RsIR11. Comparison of the mean CPM of IR transcripts among castes and between the sexes (black: female, white: male) of each caste. Young reproductives are referred to as young PQs and PKs. Mature reproductives indicate mature secondary queens and mature PKs. Error bars denote standard errors. Results of statistical analyses for each gene expression are shown in the upper right side of each graph (n.s.: not significant, *: FDR < 0.05, **: FDR < 0.01, ***: FDR < 0.001).
(EPS)

S6 Fig Expression levels of OBPs and CSPs. Comparison of the mean CPM of OBP and CSP transcripts among castes and between the sexes (black: female, white: male) of each caste. Young reproductives are referred to as young PQs and PKs. Mature reproductives indicate mature secondary queens and mature PKs. Error bars denote standard errors. Results of statistical analyses for each gene expression are shown in the upper right side of each graph (n.s.: not significant, *: FDR < 0.05, **: FDR < 0.01, ***: FDR < 0.001).
(EPS)

S1 Table RNA-seq sample statistics. Numbers in colony codes indicate the dates when the colonies were collected (e.g. colony TA130412C was collected on April 12, 2013). *: Ten individuals were pooled for each sex of worker and soldier to obtain sufficient amount of RNA, while single individuals were used for RNA extraction of the other castes.
(DOC)

S2 Table Summary results of blast homology searches. Blast homology searches of amino acid sequences in *R*. *speratus* were performed on the sequences of a grasshopper (*L*. *migratoria*), a cockroach (*P*. *americana*), a termite (*Z*. *nevadensis*), bugs (*R*. *prolixus* and *A*. *lineolatus*), a honeybee (*A*. *mellifera*), beetles (*T*. *castaneum*, *M*. *caryae*, *H*. *oblita*, and *D*. *ponderosae*), a silkworm (*B*. *mori*), mosquitoes (*A*. *gambiae* and *A*. *aegypti*), and a fruit fly (*D*. *melanogaster*).
(DOCX)

S3 Table Statistical results of differential expression levels among castes and sexes for each gene. Comparison of normalized counts per million (CPM) among castes and sexes was conducted using edgeR package. Bold letters means significant difference (FDR < 0.05). LR: likelihood ratio, FDR: false discovery rate, OR: odorant receptor, GR: gustatory receptor, IR: ionotropic receptor, OBP: odorant-binding protein, CSP: chemosensory protein.
(DOCX)

S4 Table Statistical results of age-related expression changes in male or female reproductives. Comparison of normalized counts per million (CPM) among male reproductives (alates, young and mature primary kings) or female ones (alate and young primary queens) was conducted using edgeR package. Bold letters means significant difference (FDR < 0.05). Abbreviations were referred to S3 Table.
(DOCX)
