# Supplementary material for: Caste-Specific and Sex-Specific Expression of Chemoreceptor Genes in a Termite
Source: PLoS One. 2016 Jan 13;11(1):e0146125. doi: 10.1371/journal.pone.0146125 (PMC4712011; doi:10.1371/journal.pone.0146125)
Supplement: S1 Table — Numbers in colony codes indicate the dates when the colonies were collected (e.g. colony TA130412C was collected on April 12, 2013). *: Ten individuals were pooled for each sex of worker and soldier to obtain sufficient amount of RNA, while single individuals were used for RNA extraction of the other castes. (DOC) [file pone.0146125.s007.doc]

**Table S1. RNA-seq sample statistics**.

| Colony code | Location | Sampling date | Caste | Sex | BioSample ID | # pair-reads | # mapped pair-reads (mapped rate) |
| --- | --- | --- | --- | --- | --- | --- | --- |
| HI130508N | Kitashirakawa Kogamedani, Kyoto, Kyoto | May 8, 2013 | Alate | Female | SAMD00026264 | 19779025 | 14193974 (71.76%) |
| HI130508N | Kitashirakawa Kogamedani, Kyoto, Kyoto | May 8, 2013 | Alate | Male | SAMD00026265 | 10753962 | 7230332 (67.23%) |
| HI130508N | Kitashirakawa Kogamedani, Kyoto, Kyoto | May 8, 2013 | Young primary king | Male | SAMD00026266 | 12731934 | 9772328 (76.75%) |
| HI130508N | Kitashirakawa Kogamedani, Kyoto, Kyoto | May 8, 2013 | Young primary king | Male | SAMD00026267 | 11710215 | 8524868 (72.80%) |
| HI130508N | Kitashirakawa Kogamedani, Kyoto, Kyoto | May 8, 2013 | Young primary king | Male | SAMD00026268 | 14803097 | 10370060 (70.05%) |
| HI130508N | Kitashirakawa Kogamedani, Kyoto, Kyoto | May 8, 2013 | Young primary king | Male | SAMD00026269 | 11683346 | 8499241 (72.75%) |
| HI130508N | Kitashirakawa Kogamedani, Kyoto, Kyoto | May 8, 2013 | Young primary king | Male | SAMD00026270 | 5929067 | 4183079 (70.55%) |
| HI130508N | Kitashirakawa Kogamedani, Kyoto, Kyoto | May 8, 2013 | Young primary queen | Female | SAMD00026271 | 19989225 | 15654651 (78.32%) |
| HI130508N | Kitashirakawa Kogamedani, Kyoto, Kyoto | May 8, 2013 | Young primary queen | Female | SAMD00026272 | 1419 | 951 (67.02%) |
| HI130508N | Kitashirakawa Kogamedani, Kyoto, Kyoto | May 8, 2013 | Young primary queen | Female | SAMD00026273 | 11381053 | 8335480 (73.24%) |
| HI130508N | Kitashirakawa Kogamedani, Kyoto, Kyoto | May 8, 2013 | Young primary queen | Female | SAMD00026274 | 5356648 | 3943825 (73.62%) |
| HI130508N | Kitashirakawa Kogamedani, Kyoto, Kyoto | May 8, 2013 | Young primary queen | Female | SAMD00026275 | 7032108 | 5404148 (76.85%) |
| HI130717B | Kitashirakawa Kogamedani, Kyoto, Kyoto | July 17, 2013 | Mature primary king | Male | SAMD00026276 | 7273746 | 4737937 (65.14%) |
| HI130717B | Kitashirakawa Kogamedani, Kyoto, Kyoto | July 17, 2013 | Soldier* | Female | SAMD00026277 | 6381847 | 4187391 (65.61%) |
| HI130717B | Kitashirakawa Kogamedani, Kyoto, Kyoto | July 17, 2013 | Soldier* | Male | SAMD00026278 | 14552423 | 11302911 (77.67%) |
| HI130717B | Kitashirakawa Kogamedani, Kyoto, Kyoto | July 17, 2013 | Mature secondary queen | Female | SAMD00026279 | 10709083 | 8455008 (78.95%) |
| HI130717B | Kitashirakawa Kogamedani, Kyoto, Kyoto | July 17, 2013 | Worker* | Female | SAMD00026280 | 8360113 | 6062937 (72.52%) |
| HI130717B | Kitashirakawa Kogamedani, Kyoto, Kyoto | July 17, 2013 | Worker* | Male | SAMD00026281 | 10476181 | 7610145 (72.64%) |
| TA130412C | Takaragaike Park, Kyoto, Kyoto | April 12, 2013 | Alate | Female | SAMD00026282 | 10159690 | 7549157 (74.30%) |
| TA130412C | Takaragaike Park, Kyoto, Kyoto | April 12, 2013 | Alate | Male | SAMD00026283 | 7183043 | 5429689 (75.59%) |
| TA130412C | Takaragaike Park, Kyoto, Kyoto | April 12, 2013 | Young primary king | Male | SAMD00026284 | 12535883 | 9417507 (75.12%) |
| TA130412C | Takaragaike Park, Kyoto, Kyoto | April 12, 2013 | Young primary king | Male | SAMD00026285 | 12934885 | 9624313 (74.41%) |
| TA130412C | Takaragaike Park, Kyoto, Kyoto | April 12, 2013 | Young primary king | Male | SAMD00026286 | 11984462 | 9944588 (82.98%) |
| TA130412C | Takaragaike Park, Kyoto, Kyoto | April 12, 2013 | Young primary king | Male | SAMD00026287 | 11461242 | 8359328 (72.94%) |
| TA130412C | Takaragaike Park, Kyoto, Kyoto | April 12, 2013 | Young primary king | Male | SAMD00026288 | 5829761 | 4271902 (73.28%) |
| TA130412C | Takaragaike Park, Kyoto, Kyoto | April 12, 2013 | Young primary queen | Female | SAMD00026289 | 13527803 | 10081579 (74.52%) |
| TA130412C | Takaragaike Park, Kyoto, Kyoto | April 12, 2013 | Young primary queen | Female | SAMD00026290 | 12249656 | 8859699 (72.33%) |
| TA130412C | Takaragaike Park, Kyoto, Kyoto | April 12, 2013 | Young primary queen | Female | SAMD00026291 | 9236023 | 6956045 (75.31%) |
| TA130412C | Takaragaike Park, Kyoto, Kyoto | April 12, 2013 | Young primary queen | Female | SAMD00026292 | 78715285 | 60205917 (76.49%) |
| TA130412C | Takaragaike Park, Kyoto, Kyoto | April 12, 2013 | Young primary queen | Female | SAMD00026293 | 8653119 | 6641671 (76.75%) |
| TA130412E | Takaragaike Park, Kyoto, Kyoto | April 12, 2013 | Alate | Female | SAMD00026294 | 7883685 | 5686614 (72.13%) |
| TA130412E | Takaragaike Park, Kyoto, Kyoto | April 12, 2013 | Alate | Male | SAMD00026295 | 11541753 | 8174058 (70.82%) |
| TA130412E | Takaragaike Park, Kyoto, Kyoto | April 12, 2013 | Young primary king | Male | SAMD00026296 | 16917411 | 13006656 (76.88%) |
| TA130412E | Takaragaike Park, Kyoto, Kyoto | April 12, 2013 | Young primary king | Male | SAMD00026297 | 8445096 | 6011415 (71.18%) |
| TA130412E | Takaragaike Park, Kyoto, Kyoto | April 12, 2013 | Young primary king | Male | SAMD00026298 | 17268564 | 12773720 (73.97%) |
| TA130412E | Takaragaike Park, Kyoto, Kyoto | April 12, 2013 | Young primary king | Male | SAMD00026299 | 7848570 | 5464804 (69.63%) |
| TA130412E | Takaragaike Park, Kyoto, Kyoto | April 12, 2013 | Young primary king | Male | SAMD00026300 | 270430 | 187411 (69.30%) |
| TA130412E | Takaragaike Park, Kyoto, Kyoto | April 12, 2013 | Young primary queen | Female | SAMD00026301 | 12176763 | 9049187 (74.32%) |
| TA130412E | Takaragaike Park, Kyoto, Kyoto | April 12, 2013 | Young primary queen | Female | SAMD00026302 | 7737748 | 5609812 (72.50%) |
| TA130412E | Takaragaike Park, Kyoto, Kyoto | April 12, 2013 | Young primary queen | Female | SAMD00026303 | 6442662 | 4624829 (71.78%) |
| TA130412E | Takaragaike Park, Kyoto, Kyoto | April 12, 2013 | Young primary queen | Female | SAMD00026304 | 1350130 | 990778 (73.38%) |
| TA130412E | Takaragaike Park, Kyoto, Kyoto | April 12, 2013 | Young primary queen | Female | SAMD00026305 | 4429717 | 3248551 (73.34%) |
| YO131010A | Mount Yoshidayama, Kyoto, Kyoto | October 10, 2013 | Mature primary king | Male | SAMD00026306 | 3653113 | 2810584 (76.94%) |
| YO131010A | Mount Yoshidayama, Kyoto, Kyoto | October 10, 2013 | Soldier* | Female | SAMD00026308 | 13445881 | 10051966 (74.76%) |
| YO131010A | Mount Yoshidayama, Kyoto, Kyoto | October 10, 2013 | Soldier* | Male | SAMD00026309 | 5619970 | 4228592 (75.24%) |
| YO131010A | Mount Yoshidayama, Kyoto, Kyoto | October 10, 2013 | Mature secondary queen | Female | SAMD00026307 | 4968040 | 3927669 (79.06%) |
| YO131010A | Mount Yoshidayama, Kyoto, Kyoto | October 10, 2013 | Worker* | Female | SAMD00026310 | 4209904 | 3133985 (74.44%) |
| YO131010A | Mount Yoshidayama, Kyoto, Kyoto | October 10, 2013 | Worker* | Male | SAMD00026311 | 6094843 | 4638284 (76.10%) |
| ZE130827B | Zeze Hibarigaoka, Otsu, Shiga | August 27, 2013 | Mature primary king | Male | SAMD00026312 | 18463863 | 14828901 (80.31%) |
| ZE130827B | Zeze Hibarigaoka, Otsu, Shiga | August 27, 2013 | Soldier* | Female | SAMD00026313 | 13556469 | 10157385 (74.93%) |
| ZE130827B | Zeze Hibarigaoka, Otsu, Shiga | August 27, 2013 | Soldier* | Male | SAMD00026314 | 8473771 | 6133738 (72.38%) |
| ZE130827B | Zeze Hibarigaoka, Otsu, Shiga | August 27, 2013 | Mature secondary queen | Female | SAMD00026315 | 7378019 | 5321336 (72.12%) |
| ZE130827B | Zeze Hibarigaoka, Otsu, Shiga | August 27, 2013 | Worker* | Female | SAMD00026316 | 14437965 | 9922233 (68.72%) |
| ZE130827B | Zeze Hibarigaoka, Otsu, Shiga | August 27, 2013 | Worker* | Male | SAMD00026317 | 14160514 | 9665761 (68.26%) |
| ZE130827H | Zeze Hibarigaoka, Otsu, Shiga | August 27, 2013 | Mature primary king | Male | SAMD00026318 | 14815147 | 11612552 (78.38%) |
| ZE130827H | Zeze Hibarigaoka, Otsu, Shiga | August 27, 2013 | Soldier* | Female | SAMD00026319 | 14929455 | 11295532 (75.66%) |
| ZE130827H | Zeze Hibarigaoka, Otsu, Shiga | August 27, 2013 | Soldier* | Male | SAMD00026320 | 14604843 | 10814689 (74.05%) |
| ZE130827H | Zeze Hibarigaoka, Otsu, Shiga | August 27, 2013 | Mature secondary queen | Female | SAMD00026321 | 15855406 | 12447959 (78.51%) |
| ZE130827H | Zeze Hibarigaoka, Otsu, Shiga | August 27, 2013 | Worker* | Female | SAMD00026322 | 378634 | 140224 (37.03%) |
| ZE130827H | Zeze Hibarigaoka, Otsu, Shiga | August 27, 2013 | Worker* | Male | SAMD00026323 | 9108531 | 6540449 (71.81%) |

Numbers in colony codes indicate the dates when the colonies were collected (e.g. colony TA130412C was collected on April 12, 2013). *: Ten individuals were pooled for each sex of worker and soldier to obtain sufficient amount of RNA, while single individuals were used for RNA extraction of the other castes.
