# Supplementary material for: Caste-Specific and Sex-Specific Expression of Chemoreceptor Genes in a Termite
Source: PLoS One. 2016 Jan 13;11(1):e0146125. doi: 10.1371/journal.pone.0146125 (PMC4712011; doi:10.1371/journal.pone.0146125)
Supplement: S2 Table — Blast homology searches of amino acid sequences in R. speratus were performed on the sequences of a grasshopper (L. migratoria), a cockroach (P. americana), a termite (Z. nevadensis), bugs (R. prolixus and A. lineolatus), a honeybee (A. mellifera), beetles (T. castaneum, M. caryae, H. oblita, and D. ponderosae), a silkworm (B. mori), mosquitoes (A. gambiae and A. aegypti), and a fruit fly (D. melanogaster). (DOCX) [file pone.0146125.s008.docx]

**Table S2. Summary results of blast homology searches.**

| *Reticulitermes speratus* | | | |  | Hitted genes in blastp search | | | | | |
| --- | --- | --- | --- | --- | --- | --- | --- | --- | --- | --- |
| Gene name | Accession No. | Size (aa) | Predicted ligand |  | Receptor name | Accession No. | Ligand | Organism | E−value | Identity |
| **Odorant receptor** | | | |  |  |  |  |  |  |  |
| RsOr1 | FX982902 | 102 | Unknown |  | McOR40, partial | − |  | *Megacyllene caryae* | 4E−08 | 36% |
| RsOr2 | FX982903 | 154 | Unknown |  | hypothetical protein L798_11783 | KDR14145.1 | Unknown | *Zootermopsis nevadensis* | 4E−66 | 73% |
|  |  |  |  |  | BmOr30, partial | ABK27847.1 | Unknwon | *Bombyx mori* | 3E−08 | 25% |
|  |  |  |  |  | onOr44, partial | JAA74480.1 |  | *Dendroctonus ponderosae* | 3E−18 | 33% |
| RsOr3 | FX982904 | 429 | (±)-2-methyl-1-butanol |  | hypothetical protein L798_14033 | KDR11401.1 | Unknown | *Zootermopsis nevadensis* | 8E−104 | 38% |
|  |  |  |  |  | AGAP002558−PA (OR40) | EAA08058.2 | Unknown | *Anopheles gambiae* | 1E−24 | 26% |
|  |  |  |  |  | olfactory receptor putative, partial | XP_001651429.1 | Unknown | *Aedes aegypti* | 8E−22 | 25% |
|  |  |  |  |  | BmOr63 | NP_001166620.1 | Unknown | *Bombyx mori* | 2E−32 | 25% |
|  |  |  |  |  | TcOr24 | EFA10799.1 |  | *Tribolium castaneum* | 1E−32 | 23% |
|  |  |  |  |  | McOR3 | − | 2-methyl-1-butanol | *Megacyllene caryae* | 7E−32 | 25% |
|  |  |  |  |  | onOr6 | JAA74487.1 | Unknown | *Dendroctonus ponderosae* | 2E−22 | 22% |
|  |  |  |  |  | AmOR53 | NP_001229907.1 | Unknown | *Apis melifera* | 7E−13 | 25% |
|  |  |  |  |  | LmigOr4 | AGK25860.1 |  | *Locusta migaratoria* | 7E−34 | 26% |
| RsOr4 | FX982905 | 334 | Unknown |  | hypothetical protein L798_03923 | KDR06653.1 | Unknown | *Zootermopsis nevadensis* | 2E−179 | 71% |
| RsOr5 | FX982906 | 469 | Unknown |  | hypothetical protein L798_13613 | KDR11960.1 | Unknown | *Zootermopsis nevadensis* | 3E−156 | 50% |
| RsOr6 | FX982907 | 412 | Unknown |  | hypothetical protein L798_07577, partial | KDQ84123.1 | Unknown | *Zootermopsis nevadensis* | 4E−61 | 38% |
|  |  |  |  |  | olfactory receptor | BAH66346.1 | Unknown | *Bombyx mori* | 4E−16 | 29% |
|  |  |  |  |  | McOR24, partial | − |  | *Megacyllene caryae* | 7E−08 | 26% |
|  |  |  |  |  | AmOR57 | NP_001229910.1 | Unknown | *Apis melifera* | 2E−14 | 24% |
| RsOr7 | FX982908 | 407 | Unknown |  | hypothetical protein L798_03924 | KDR06654.1 | Unknown | *Zootermopsis nevadensis* | 0E+00 | 70% |
|  |  |  |  |  | AmOR115 | NP_001229918.1 | Unknown | *Apis melifera* | 3E−17 | 23% |
| RsOr8 | FX982909 | 212 | Unknown |  | McOR23 | − | Unknown | *Megacyllene caryae* | 2E−08 | 25% |
|  |  |  |  |  | AmOR55 | NP_001229908.1 | Unknown | *Apis melifera* | 3E−13 | 27% |
|  |  |  |  |  | AmOR56 | NP_001229909.1 | Unknown | *Apis melifera* | 3E−13 | 27% |
| RsOr9 | FX982910 | 462 | Unknown |  | hypothetical protein L798_02806 | KDR21745.1 | Unknown | *Zootermopsis nevadensis* | 3E−75 | 70% |
|  |  |  |  |  | olfactory receptor | BAH66322.1 | Unknown | *Bombyx mori* | 8E−15 | 30% |
|  |  |  |  |  | onOr5 | JAA74473.1 | Unknown | *Dendroctonus ponderosae* | 4E−16 | 27% |
|  |  |  |  |  | AmOR63 | AHJ37469.1 | Unknown | *Apis melifera* | 8E−17 | 27% |
| RsOr10 | FX982911 | 492 | Ethylbutyrate |  | hypothetical protein L798_06522 | KDR19479.1 | Unknown | *Zootermopsis nevadensis* | 8E−110 | 39% |
|  |  |  |  |  | Or22a | AAF51364.1 | Ethylbutyrate | *Drosophila melanogaster* | 4E-09 | 25% |
|  |  |  |  |  | BmOr11 | NP_001166604.1 | Unknown | *Bombyx mori* | 7E−12 | 31% |
|  |  |  |  |  | McOR25, partial | − | Unknown | *Megacyllene caryae* | 1E−98 | 25% |
|  |  |  |  |  | onOr15 | JAA74461.1 | Unknown | *Dendroctonus ponderosae* | 8E−09 | 25% |
| RsOr11 | FX982912 | 236 | Unknown |  | olfactory receptor | BAH66342.1 | Unknown | *Bombyx mori* | 2E−23 | 33% |
|  |  |  |  |  | McOR30 | − | Unknown | *Megacyllene caryae* | 1E−10 | 26% |
|  |  |  |  |  | onOr11, partial | JAA74516.1 | Unknown | *Dendroctonus ponderosae* | 5E−19 | 31% |
|  |  |  |  |  | LmigOr2 | AFL03413.1 | Unknown | *Locusta migaratoria* | 4E−14 | 29% |
| RsOr12 | FX982913 | 225 | Unknown |  | olfactory receptor | BAH66361.1 | Unknown | *Bombyx mori* | 9E−11 | 24% |
|  |  |  |  |  | McOR18 | − | Unknown | *Megacyllene caryae* | 8E−14 | 29% |
|  |  |  |  |  | onOr9, partial | JAA74460.1 | Unknown | *Dendroctonus ponderosae* | 3E−12 | 24% |
| RsORCO  (RsOr13) | FX982914 | 473 | Orco |  | gustatory and odorant receptor 7 | KDR12002.1 | ORCO | *Zootermopsis nevadensis* | 0E+00 | 91% |
|  |  |  |  |  | DmOR83b | AAT71306.1 | ORCO | *Drosophila melanogaster* | 0E+00 | 72% |
|  |  |  |  |  | AgOR83b | AAX14774.1 | ORCO | *Anopheles gambiae* | 0E+00 | 73% |
|  |  |  |  |  | AaOR7 | Q178U6.1 | ORCO | *Aedes aegypti* | 0E+00 | 72% |
|  |  |  |  |  | BmOR2 | NP_001037060.1 | ORCO | *Bombyx mori* | 0E+00 | 59% |
|  |  |  |  |  | PREDICTED: odorant receptor coreceptor | XP_008194693.1 | ORCO | *Tribolium castaneum* | 0E+00 | 64% |
|  |  |  |  |  | McOR1 | − | ORCO | *Megacyllene caryae* | 0E+00 | 64% |
|  |  |  |  |  | hypothetical protein D910_00196, partial | ERL95741.1 | ORCO | *Dendroctonus ponderosae* | 0E+00 | 64% |
|  |  |  |  |  | AmOR2 | AHJ37468.1 | ORCO | *Apis melifera* | 0E+00 | 60% |
|  |  |  |  |  | LmigOrco, partial | AEX28370.1 | ORCO | *Locusta migaratoria* | 0E+00 | 63% |
| RsOr14 | FX982915 | 360 | Unknown |  | hypothetical protein L798_03321 | KDR21850.1 | Unknown | *Zootermopsis nevadensis* | 2E−163 | 64% |
|  |  |  |  |  | AGAP001912−PA | EAA01023.2 | Unknown | *Anopheles gambiae* | 8E−21 | 32% |
| RsOr15 | FX982916 | 241 | Unknown |  | hypothetical protein L798_12093 | KDR13797.1 | Unknown | *Zootermopsis nevadensis* | 3E−119 | 69% |
|  |  |  |  |  | BmOr18 | NP_001166895.1 | Unknown | *Bombyx mori* | 4E−12 | 30% |
| RsOr16 | FX982917 | 120 | Unknown |  | Putative odorant receptor 85f | KDR17509.1 | Unknown | *Zootermopsis nevadensis* | 3E−58 | 94% |
| RsOr17 | FX982918 | 219 | Unknown |  | hypothetical protein L798_15021 | KDR11214.1 | Unknown | *Zootermopsis nevadensis* | 0E+00 | 69% |
|  |  |  |  |  | BmOr47, partial | ABK27854.1 | Unknown | *Bombyx mori* | 6E−24 | 36% |
|  |  |  |  |  | PREDICTED: OR1−like | XP_008190424.1 | Unknown | *Tribolium castaneum* | 1E−26 | 33% |
|  |  |  |  |  | McOR37, partial | − | Unknown | *Megacyllene caryae* | 2E−24 | 31% |
|  |  |  |  |  | onOr16 | JAA74512.1 | Unknown | *Dendroctonus ponderosae* | 4E−25 | 34% |
| RsOr18 | FX982919 | 482 | Unknown |  | hypothetical protein L798_07852 | KDR17468.1 | Unknown | *Zootermopsis nevadensis* | 0E+00 | 53% |
|  |  |  |  |  | BmOr10 | NP_001104819.1 | Unknown | *Bombyx mori* | 1E−19 | 26% |
|  |  |  |  |  | McOR36, partial | − | Unknown | *Megacyllene caryae* | 2E−20 | 33% |
|  |  |  |  |  | onOr3 | JAA74476.1 | Unknown | *Dendroctonus ponderosae* | 1E−13 | 25% |
|  |  |  |  |  | AmOR81, isoform 1, partial | AHJ37470.1 | Unknown | *Apis melifera* | 4E−17 | 22% |
| RsOr19 | FX982920 | 125 | Unknown |  | Or2 | KDR11039.1 | Unknown | *Zootermopsis nevadensis* | 2E−45 | 84% |
| RsOr20 | FX982921 | 294 | Unknown |  | hypothetical protein L798_14624 | KDR11040.1 | Unknown | *Zootermopsis nevadensis* | 5E−159 | 78% |
|  |  |  |  |  | BmOr29, partial | DAA05985.1 | Unknown | *Bombyx mori* | 1E−07 | 21% |
| RsOr21 | FX982922 | 200 | Unknown |  | hypothetical protein L798_15004 | KDR10321.1 | Unknown | *Zootermopsis nevadensis* | 4E−129 | 48% |
|  |  |  |  |  | BmOr24 | NP_001155300.1 | Unknown | *Bombyx mori* | 5E−16 | 31% |
|  |  |  |  |  | TcOr14 | EFA09245.1 | Unknown | *Tribolium castaneum* | 9E−22 | 33% |
|  |  |  |  |  | McOR56, partial | − | Unknown | *Megacyllene caryae* | 7E−17 | 30% |
|  |  |  |  |  | onOr30 | JAA74510.1 | Unknown | *Dendroctonus ponderosae* | 5E−17 | 29% |
|  |  |  |  |  | AmOR27 | NP_001229898.1 | Unknown | *Apis melifera* | 2E−14 | 28% |
| RsOr22 | FX982923 | 383 | Unknown |  | hypothetical protein L798_06230 | KDR19176.1 | Unknown | *Zootermopsis nevadensis* | 8E−170 | 61% |
| **Gustatory receptor** | | | |  |  |  |  |  |  |  |
| RsGR1 | FX982924 | 326 | Glycerol |  | GR64e | P83296.2 | Glycerol | *Drosophila melanogaster* | 1E−62 | 36% |
|  |  |  |  |  | GR16 | EFA04722.1 | Unknown | *Tribolium castaneum* | 5E−35 | 27% |
| RsGR2 | FX982925 | 430 | CO_2_ receptor A |  | hypothetical protein L798_00586 | KDR09715.1 | Unknown | *Zootermopsis nevadensis* | 2E−100 | 44% |
|  |  |  |  |  | GR63a | Q9VZL7.1 | CO_2_ | *Drosophila melanogaster* | 1E−54 | 31% |
| RsGR3 | FX982926 | 349 | Unknown |  | PREDICTED: GR64f−like | XP_008193175.1 | Unknown | *Tribolium castaneum* | 7E−33 | 41% |
| RsGR4 | FX982927 | 472 | Unknown |  | GR19 | EFA04725.1 | Unknown | *Tribolium castaneum* | 8E−42 | 32% |
| RsGR5 | FX982928 | 423 | Trehalose, Melezitose, m-glucoside |  | Gr64f | P83297.2 | Trehalose, Melezitose, m-glucocide | *Drosophila melanogaster* | 3E−80 | 37% |
|  |  |  |  |  | Gr11 | EFA04717.1 | Unknown | *Tribolium castaneum* | 7E−84 | 37% |
| RsGR6 | FX982929 | 409 | CO_2_ receptor B |  | GR21a | Q9VPT1.3 | CO_2_ | *Drosophila melanogaster* | 2E−42 | 23% |
|  |  |  |  |  | Gr2 | EFA02924.1 | Unknown | *Tribolium castaneum* | 1E−51 | 30% |
| RsGR7 | FX982930 | 259 | Trehalose |  | Gustatory receptor trehalose 1 | KDR12697.1 | Trehalose | *Zootermopsis nevadensis* | 1E−159 | 83% |
| **Ionotropic receptor** | | | |  |  |  |  |  |  |  |
| RsIR1 | FX982931 | 640 | Butyrate, Butyraldehyde, Propionate, Acetate |  | IR75a | AAF49300.2 | Butyrate, Butyraldehyde, Propionate, Acetate | *Drosophila melanogaster* | 1E−30 | 24% |
| RsIR2 | FX982932 | 474 | Unknown |  | IR100a | AAF57202.2 | Unknown | *Drosophila melanogaster* | 1E−42 | 27% |
| RsIR3 | FX982933 | 523 | Unknown |  | IR21a | AAF51569.2 | Unknown | *Drosophila melanogaster* | 9E−95 | 47% |
| RsIR4 | FX982934 | 361 | Unknown |  | IR93a | AAF55817.3 | Unknown | *Drosophila melanogaster* | 2E−81 | 38% |
| RsIR5 | FX982935 | 534 | Ammonia, Dimethylamine, Diaminobutane, Phenylethylamine |  | IR76b | AAF49071.1 | Ammonia, Dimethylamine, Diaminobutane, Phenylethylamine | *Drosophila melanogaster* | 8E−122 | 40% |
| RsIR6 | FX982936 | 493 | butyrate/butyraldehyde/propionate/acetate |  | IR75a | ADQ74919.1 | butyrate/butyraldehyde/propionate/acetate | *Drosophila melanogaster* | 5E−31 | 22% |
| RsIR7 | FX982937 | 604 | Unknown |  | IR68a, isoform A | AAF50075.2 | Unknown | *Drosophila melanogaster* | 8E−121 | 37% |
| RsIR8 | FX982938 | 443 | Ammonia, Dimethylamine, Diaminobutane, Phenylethylamine |  | IR41a | AAS64776.4 | Ammonia, Dimethylamine, Diaminobutane, Phenylethylamine | *Drosophila melanogaster* | 4E−65 | 32% |
| RsIR9 | FX982939 | 618 | IR co−receptor A |  | IR8a | AAF46470.2 | co−receptor | *Drosophila melanogaster* | 2E−143 | 41% |
| RsIR10 | FX982940 | 617 | IR co−receptor B |  | IR25a | ADU79032.1 | co−receptor | *Drosophila melanogaster* | 0E+00 | 67% |
| RsIR11 | FX982941 | 605 | pyrrolidine |  | IR75d | AAF49210.2 | pyrrolidine | *Drosophila melanogaster* | 1E−71 | 33% |
| RsIR12 | FX982942 | 550 | Phenylacetic acid |  | IR84a | ADU79034.1 | Phenylacetic acid | *Drosophila melanogaster* | 2E−40 | 28% |
| **Odorant−binding protein** | | | |  |  |  |  |  |  |  |
| RsOBP1 | FX982943 | 267 | Unknown |  | putative odorant binding protein | JAA75174.1 | Unknown | *Rhodnius prolixus* | 6E−98 | 56% |
| RsOBP2 | FX982944 | 163 | Unknown |  | odorant binding protein 11 | EFA05695.1 | Unknown | *Tribolium castaneum* | 9E−42 | 47% |
| RsOBP3 | FX982945 | 200 | Unknown |  | A5 | AAC46472.1 | Unknown | *Drosophila melanogaster* | 2E−51 | 40% |
| RsOBP4 | FX982946 | 151 | Unknown |  | odorant−binding protein | ACI30685.1 | Unknown | *Periplaneta americana* | 2E−56 | 76% |
| RsOBP5 | FX982947 | 263 | Unknown |  | putative odorant binding protein | JAA75367.1 | Unknown | *Rhodnius prolixus* | 3E−118 | 62% |
| RsOBP6 | FX982948 | 284 | Unknown |  | putative odorant binding protein | JAA75606.1 | Unknown | *Rhodnius prolixus* | 3E−114 | 64% |
| RsOBP7 | FX982949 | 178 | General odor |  | General odorant−binding protein 70 | Q7Q5L4.2 | Unknown | *Anopheles gambiae* | 2E−38 | 36% |
| RsOBP8 | FX982950 | 253 | Unknown |  | putative odorant binding protein | JAA75245.1 | Unknown | *Rhodnius prolixus* | 3E−45 | 34% |
| RsOBP9 | FX982951 | 264 | Unknown |  | putative odorant binding protein, partial | JAA77291.1 | Unknown | *Rhodnius prolixus* | 4E−48 | 37% |
| **Chemosensory protein** | | | |  |  |  |  |  |  |  |
| RsCSP1 | FX982952 | 144 | α-ionone, β-ionone, cinnarnaldehyde |  | chemosensory protein 10 | ABH88203.1 | Unknown | *Bombyx mori* | 7E−37 | 46% |
|  |  |  |  |  | Chemosensory protein 18 | EFA07570.1 | Unknown | *Tribolium castaneum* | 9E−40 | 52% |
|  |  |  |  |  | Chemosensory protein 20 | ABH88193.1 | Unknown | *Tribolium castaneum* | 9E−40 | 52% |
|  |  |  |  |  | HoblCSP1 | − | α-ionone, β-ionone, cinnarnaldehyde | *Holotrichia oblita* | 3E−29 | 44% |
|  |  |  |  |  | AlinCSP3 | − | Unknown | *Adelphocoris lineolatus* | 9E−36 | 70% |
| RsCSP2 | FX982953 | 128 | Unknown |  | chemosensory protein 16 | ABH88209.1 | Unknown | *Bombyx mori* | 1E−42 | 67% |
|  |  |  |  |  | chemosensory protein 17 | EEZ99322.1 | Unknown | *Tribolium castaneum* | 1E−43 | 64% |
| RsCSP3 | FX982954 | 136 | Unknown |  | chemosensory protein 11 | ABH88204.1 | Unknown | *Bombyx mori* | 1E−31 | 42% |

Blast homology searches of amino acid sequences in *Reticulitermes speratus* were performed on the sequences of a grasshopper (*Locusta migratoria*), a cockroach (*Periplaneta americana*), a termite (*Zootermopsis nevadensis*), bugs (*Rhodnius prolixus* and *Adelphocoris lineolatus*), a honeybee (*Apis mellifera*), beetles (*Tribolium castaneum*, *Megacyllene caryae*, *Holotrichis oblita*, and *Dendroctonus ponderosae*), a silkworm (*Bombyx mori*), mosquitoes (*Anopheles gambiae* and *Aedes aegypti*), and a fruit fly (*Drosophila melanogaster*).
