# Supplementary material for: Caste-Specific and Sex-Specific Expression of Chemoreceptor Genes in a Termite
Source: PLoS One. 2016 Jan 13;11(1):e0146125. doi: 10.1371/journal.pone.0146125 (PMC4712011; doi:10.1371/journal.pone.0146125)
Supplement: S3 Table — Comparison of normalized counts per million (CPM) among castes and sexes was conducted using edgeR package. Bold letters means significant difference (FDR < 0.05). LR: likelihood ratio, FDR: false discovery rate, OR: odorant receptor, GR: gustatory receptor, IR: ionotropic receptor, OBP: odorant-binding protein, CSP: chemosensory protein. (DOCX) [file pone.0146125.s009.docx]

**Table S3. Statistical results of differential expression levels among castes and sexes for each gene.**

| Gene | Predicted ligand | Caste | | |  | Sex | | |  | Caste × Sex | | |
| --- | --- | --- | --- | --- | --- | --- | --- | --- | --- | --- | --- | --- |
|  |  | LR | P | FDR |  | LR | P | FDR |  | LR | P | FDR |
| RsOr1 | Unknown | 5.620 | 0.230 | 1.000 |  | 0.000 | 1.000 | 1.000 |  | 8.780 | 0.067 | 1.000 |
| RsOr2 | Unknown | **28.400** | **0.000** | **0.000** |  | 0.066 | 0.797 | 1.000 |  | 10.800 | 0.291 | 0.745 |
| RsOr3 | (±)-2-methyl-1-butanol | **42.500** | **0.000** | **0.000** |  | 0.000 | 1.000 | 1.000 |  | 5.430 | 0.246 | 1.000 |
| RsOr4 | Unknown | 9.140 | 0.058 | 0.347 |  | 0.000 | 1.000 | 1.000 |  | 9.980 | 0.041 | 0.922 |
| RsOr5 | Unknown | **73.000** | **0.000** | **0.000** |  | 0.000 | 1.000 | 1.000 |  | 13.000 | 0.012 | 0.402 |
| RsOr6 | Unknown | **42.700** | **0.000** | **0.000** |  | 0.001 | 0.970 | 1.000 |  | 6.040 | 0.197 | 1.000 |
| RsOr7 | Unknown | **70.100** | **0.000** | **0.000** |  | 7.850 | 0.005 | 0.785 |  | 1.980 | 0.740 | 1.000 |
| RsOr8 | Unknown | **261.000** | **0.000** | **0.000** |  | **16.900** | **0.000** | **0.032** |  | **37.800** | **0.000** | **0.000** |
| RsOr9 | Unknown | **75.900** | **0.000** | **0.000** |  | 2.300 | 0.129 | 1.000 |  | 7.9200 | 0.095 | 1.000 |
| RsOr10 | Ethyl butyrate | **70.700** | **0.000** | **0.000** |  | 7.490 | 0.006 | 0.847 |  | **21.800** | **0.000** | **0.022** |
| RsOr11 | Unknown | 10.000 | 0.040 | 0.253 |  | 0.032 | 0.859 | 1.000 |  | 2.700 | 0.609 | 1.000 |
| RsOr12 | Unknown | **30.600** | **0.000** | **0.000** |  | 0.225 | 0.636 | 1.000 |  | 14.700 | 0.005 | 0.233 |
| RsORCO | (ORCO) | **201.000** | **0.000** | **0.000** |  | 11.500 | 0.001 | 0.250 |  | 13.900 | 0.008 | 0.305 |
| RsOr14 | Unknown | **51.400** | **0.000** | **0.000** |  | 0.149 | 0.699 | 1.000 |  | 15.700 | 0.003 | 0.171 |
| RsOr15 | Unknown | **22.000** | **0.000** | **0.002** |  | 6.510 | 0.011 | 1.000 |  | 17.100 | 0.002 | 0.108 |
| RsOr16 | Unknown | **30.000** | **0.000** | **0.000** |  | 1.030 | 0.311 | 1.000 |  | 4.020 | 0.403 | 1.000 |
| RsOr17 | Unknown | 12.000 | 0.017 | 0.124 |  | 1.450 | 0.229 | 1.000 |  | **21.700** | **0.000** | **0.022** |
| RsOr18 | Unknown | **158.000** | **0.000** | **0.000** |  | 0.850 | 0.357 | 1.000 |  | **26.500** | **0.000** | **0.004** |
| RsOr19 | Unknown | 8.750 | 0.068 | 0.397 |  | 0.437 | 0.508 | 1.000 |  | 17.200 | 0.002 | 0.107 |
| RsOr20 | Unknown | 12.000 | 0.017 | 0.122 |  | 2.690 | 0.101 | 1.000 |  | **19.700** | **0.001** | **0.046** |
| RsOr21 | Unknown | **126.000** | **0.000** | **0.000** |  | 0.008 | 0.930 | 1.000 |  | 3.760 | 0.439 | 1.000 |
| RsOr22 | Unknown | **107.000** | **0.000** | **0.000** |  | **22.900** | **0.000** | **0.003** |  | **27.700** | **0.000** | **0.002** |
| RsGr1 | Trehalose / Melezitose / m-glucoside | 11.100 | 0.026 | 0.174 |  | 2.740 | 0.098 | 1.000 |  | **19.700** | **0.001** | **0.045** |
| RsGr2 | CO_2_ | **54.600** | **0.000** | **0.000** |  | 0.000 | 1.000 | 1.000 |  | 0.150 | 0.997 | 1.000 |
| RsGr3 | Unknown | 5.940 | 0.203 | 0.959 |  | 14.900 | 0.000 | 0.072 |  | 6.010 | 0.198 | 1.000 |
| RsGr4 | Unknown | **65.300** | **0.000** | **0.000** |  | 5.270 | 0.022 | 1.000 |  | **101.000** | **0.000** | **0.000** |
| RsGr5 | Glycerol | **155.000** | **0.000** | **0.000** |  | **23.500** | **0.000** | **0.002** |  | **44.200** | **0.000** | **0.000** |
| RsGr6 | CO_2_ | **26.500** | **0.000** | **0.000** |  | 0.646 | 0.422 | 1.000 |  | 17.800 | 0.001 | 0.087 |
| RsGr7 | Trehalose | **330.000** | **0.000** | **0.000** |  | **61.100** | **0.000** | **0.000** |  | **154.000** | **0.000** | **0.000** |
| RsIR1 | Butyrate / Butyraldehyde / Propionate / Acetate | **120.000** | **0.000** | **0.000** |  | 1.380 | 0.240 | 1.000 |  | 16.500 | 0.002 | 0.132 |
| RsIR2 | Unknown | 8.370 | 0.079 | 0.454 |  | 2.730 | 0.099 | 1.000 |  | 6.480 | 0.166 | 1.000 |
| RsIR3 | Unknown | **56.800** | **0.000** | **0.000** |  | 4.580 | 0.032 | 1.000 |  | 2.810 | 0.591 | 1.000 |
| RsIR4 | Unknown | 9.950 | 0.041 | 0.261 |  | 2.150 | 0.143 | 1.000 |  | 12.800 | 0.012 | 0.425 |
| RsIR5 | Ammonia / Dimethylamine / Diaminobutane /  Phenylethylamine | **50.900** | **0.000** | **0.000** |  | 3.980 | 0.046 | 1.000 |  | 6.690 | 0.153 | 1.000 |
| RsIR6 | Butyrate / Butyraldehyde / Propionate / Acetate | **19.700** | **0.001** | **0.006** |  | 0.000 | 1.000 | 1.000 |  | 17.200 | 0.002 | 0.106 |
| RsIR7 | Unknown | **42.300** | **0.000** | **0.000** |  | 0.000 | 0.984 | 1.000 |  | 7.830 | 0.098 | 1.000 |
| RsIR8 | Ammonia / Dimethylamine / Diaminobutane /  Phenylethylamine | **31.900** | **0.000** | **0.000** |  | 0.036 | 0.850 | 1.000 |  | 6.310 | 0.177 | 1.000 |
| RsIR9 | IR8a_co-receptor | **108.000** | **0.000** | **0.000** |  | 0.310 | 0.578 | 1.000 |  | 6.280 | 0.179 | 1.000 |
| RsIR10 | IR25a_co-receptor | **113.000** | **0.000** | **0.000** |  | 9.140 | 0.003 | 0.545 |  | **32.800** | **0.000** | **0.000** |
| RsIR11 | Pyrrolidine | **114.000** | **0.000** | **0.000** |  | 10.400 | 0.001 | 0.359 |  | **37.000** | **0.000** | **0.000** |
| RsIR12 | Phenylacetic acid | **47.900** | **0.000** | **0.000** |  | 4.650 | 0.031 | 1.000 |  | 13.500 | 0.009 | 0.338 |
| RsOBP1 | Unknown | **157.000** | **0.000** | **0.000** |  | 1.390 | 0.239 | 1.000 |  | 2.390 | 0.664 | 1.000 |
| RsOBP2 | Unknown | **50.500** | **0.000** | **0.000** |  | 0.599 | 0.439 | 1.000 |  | 7.400 | 0.116 | 1.000 |
| RsOBP3 | Unknown | **27.500** | **0.000** | **0.000** |  | 0.036 | 0.849 | 1.000 |  | 1.750 | 0.782 | 1.000 |
| RsOBP4 | Unknown | **22.200** | **0.000** | **0.002** |  | 0.053 | 0.817 | 1.000 |  | 1.970 | 0.742 | 1.000 |
| RsOBP5 | Unknown | **36.200** | **0.000** | **0.000** |  | 0.843 | 0.358 | 1.000 |  | 10.800 | 0.029 | 0.741 |
| RsOBP6 | Unknown | **82.100** | **0.000** | **0.000** |  | 2.890 | 0.089 | 1.000 |  | 7.380 | 0.117 | 1.000 |
| RsOBP7 | General odor | **87.700** | **0.000** | **0.000** |  | 3.380 | 0.066 | 1.000 |  | **93.800** | **0.000** | **0.000** |
| RsOBP8 | Unknown | **121.000** | **0.000** | **0.000** |  | 0.488 | 0.485 | 1.000 |  | 10.700 | 0.031 | 0.771 |
| RsOBP9 | Unknown | **310.000** | **0.000** | **0.000** |  | 6.480 | 0.011 | 1.000 |  | **20.700** | **0.000** | **0.032** |
| RsCSP1 | α-ionone etc. | **177.000** | **0.000** | **0.000** |  | 1.010 | 0.315 | 1.000 |  | 8.180 | 0.085 | 1.000 |
| RsCSP2 | Unknown | **46.900** | **0.000** | **0.000** |  | 5.350 | 0.021 | 1.000 |  | **31.400** | **0.000** | **0.001** |
| RsCSP3 | Unknown | **250.000** | **0.000** | **0.000** |  | 0.122 | 0.727 | 1.000 |  | **24.900** | **0.000** | **0.007** |

Comparison of normalized counts per million (CPM) among castes and sexes was conducted using edgeR package. Bold letters means significant difference (FDR < 0.05). LR: likelihood ratio, FDR: false discovery rate, OR: odorant receptor, GR: gustatory receptor, IR: ionotropic receptor, OBP: odorant-binding protein, CSP: chemosensory protein.
