# Supplementary material for: Caste-Specific and Sex-Specific Expression of Chemoreceptor Genes in a Termite
Source: PLoS One. 2016 Jan 13;11(1):e0146125. doi: 10.1371/journal.pone.0146125 (PMC4712011; doi:10.1371/journal.pone.0146125)
Supplement: S4 Table — Comparison of normalized counts per million (CPM) among male reproductives (alates, young and mature primary kings) or female ones (alate and young primary queens) was conducted using edgeR package. Bold letters means significant difference (FDR < 0.05). Abbreviations were referred to S3 Table. (DOCX) [file pone.0146125.s010.docx]

**Table S4. Statistical results of age-related expression changes in male or female reproductives.**

| Gene |  | Male reproductives | | |  | Female reproductives | | |
| --- | --- | --- | --- | --- | --- | --- | --- | --- |
|  |  | LR | P | FDR |  | LR | P | FDR |
| RsOr1 |  | 4.950 | 0.084 | 0.608 |  | 0.707 | 0.400 | 1.000 |
| RsOr2 |  | **11.100** | **0.004** | **0.047** |  | 9.190 | 0.002 | 0.057 |
| RsOr3 |  | **13.800** | **0.001** | **0.015** |  | **9.900** | **0.002** | **0.041** |
| RsOr4 |  | 1.480 | 0.476 | 1.000 |  | 0.872 | 0.350 | 1.000 |
| RsOr5 |  | **16.200** | **0.000** | **0.005** |  | **13.900** | **0.000** | **0.007** |
| RsOr6 |  | 2.440 | 0.295 | 1.000 |  | 6.340 | 0.012 | 0.192 |
| RsOr7 |  | 0.626 | 0.731 | 1.000 |  | 0.996 | 0.318 | 1.000 |
| RsOr8 |  | **310.000** | **0.000** | **0.000** |  | 2.230 | 0.136 | 0.998 |
| RsOr9 |  | **36.300** | **0.000** | **0.000** |  | 5.230 | 0.022 | 0.303 |
| RsOr10 |  | **59.500** | **0.000** | **0.000** |  | 5.680 | 0.017 | 0.246 |
| RsOr11 |  | 1.510 | 0.470 | 1.000 |  | 0.611 | 0.434 | 1.000 |
| RsOr12 |  | **29.300** | **0.000** | **0.000** |  | **18.000** | **0.000** | **0.001** |
| RsORCO |  | **227.000** | **0.000** | **0.000** |  | **77.800** | **0.000** | **0.000** |
| RsOr14 |  | 4.720 | 0.094 | 0.664 |  | 3.880 | 0.049 | 0.535 |
| RsOr15 |  | 3.750 | 0.154 | 0.901 |  | **11.300** | **0.000** | **0.023** |
| RsOr16 |  | **19.600** | **0.000** | **0.001** |  | 7.650 | 0.006 | 0.109 |
| RsOr17 |  | **15.500** | **0.000** | **0.007** |  | 0.001 | 0.970 | 1.000 |
| RsOr18 |  | 2.710 | 0.258 | 1.000 |  | 0.182 | 0.670 | 1.000 |
| RsOr19 |  | **26.400** | **0.000** | **0.000** |  | 0.700 | 0.403 | 1.000 |
| RsOr20 |  | 3.010 | 0.222 | 1.000 |  | 2.610 | 0.106 | 0.842 |
| RsOr21 |  | **114.000** | **0.000** | **0.000** |  | **104.000** | **0.000** | **0.000** |
| RsOr22 |  | 4.520 | 0.104 | 0.717 |  | 1.560 | 0.212 | 1.000 |
| RsGr1 |  | **13.000** | **0.001** | **0.020** |  | 1.620 | 0.204 | 1.000 |
| RsGr2 |  | 2.790 | 0.248 | 1.000 |  | **14.000** | **0.000** | **0.006** |
| RsGr3 |  | **27.900** | **0.000** | **0.000** |  | 0.599 | 0.439 | 1.000 |
| RsGr4 |  | **35.600** | **0.000** | **0.000** |  | 0.011 | 0.915 | 1.000 |
| RsGr5 |  | **62.300** | **0.000** | **0.000** |  | **13.600** | **0.000** | **0.008** |
| RsGr6 |  | 8.480 | 0.014 | 0.146 |  | 0.368 | 0.544 | 1.000 |
| RsGr7 |  | 3.140 | 0.208 | 1.000 |  | **88.400** | **0.000** | **0.000** |
| RsIR1 |  | 1.280 | 0.528 | 1.000 |  | 7.430 | 0.006 | 0.119 |
| RsIR2 |  | 6.150 | 0.046 | 0.378 |  | 3.370 | 0.066 | 0.597 |
| RsIR3 |  | **15.800** | **0.000** | **0.006** |  | **28.100** | **0.000** | **0.000** |
| RsIR4 |  | **15.600** | **0.000** | **0.006** |  | 3.660 | 0.056 | 0.535 |
| RsIR5 |  | **34.400** | **0.000** | **0.000** |  | **2.830** | **0.000** | **0.000** |
| RsIR6 |  | 10.300 | 0.006 | 0.067 |  | 3.940 | 0.047 | 0.520 |
| RsIR7 |  | 6.740 | 0.035 | 0.298 |  | 9.320 | 0.002 | 0.054 |
| RsIR8 |  | 2.770 | 0.251 | 1.000 |  | 4.110 | 0.043 | 0.483 |
| RsIR9 |  | **74.100** | **0.000** | **0.000** |  | **59.700** | **0.000** | **0.000** |
| RsIR10 |  | **18.000** | **0.000** | **0.002** |  | **48.900** | **0.000** | **0.000** |
| RsIR11 |  | **270.000** | **0.000** | **0.000** |  | 5.170 | 0.023 | 0.311 |
| RsIR12 |  | 3.950 | 0.139 | 0.860 |  | **52.500** | **0.000** | **0.000** |
| RsOBP1 |  | **84.800** | **0.000** | **0.000** |  | **67.700** | **0.000** | **0.000** |
| RsOBP2 |  | 2.790 | 0.247 | 1.000 |  | 5.980 | 0.015 | 0.220 |
| RsOBP3 |  | **18.400** | **0.000** | **0.002** |  | 7.400 | 0.007 | 0.121 |
| RsOBP4 |  | **12.700** | **0.002** | **0.024** |  | **14.400** | **0.000** | **0.005** |
| RsOBP5 |  | **18.000** | **0.000** | **0.002** |  | 0.054 | 0.817 | 1.000 |
| RsOBP6 |  | 9.740 | 0.008 | 0.086 |  | **23.300** | **0.000** | **0.000** |
| RsOBP7 |  | **24.700** | **0.000** | **0.000** |  | **16.600** | **0.000** | **0.002** |
| RsOBP8 |  | 1.590 | 0.452 | 1.000 |  | 6.340 | 0.012 | 0.191 |
| RsOBP9 |  | **84.900** | **0.000** | **0.000** |  | **40.200** | **0.000** | **0.000** |
| RsCSP1 |  | 0.949 | 0.622 | 1.000 |  | 0.574 | 0.449 | 1.000 |
| RsCSP2 |  | **19.900** | **0.000** | **0.000** |  | 3.010 | 0.083 | 0.703 |
| RsCSP3 |  | **95.200** | **0.000** | **0.000** |  | **59.700** | **0.000** | **0.000** |

Comparison of normalized counts per million (CPM) among male reproductives (alates, young and mature primary kings) or female ones (alate and young primary queens) was conducted using edgeR package. Bold letters means significant difference (FDR < 0.05). Abbreviations were referred to Table S2.
